# Supplementary material for: Arl13b Regulates Breast Cancer Cell Migration and Invasion by Controlling Integrin-Mediated Signaling
Source: Cancers (Basel). 2019 Sep 29;11(10):1461. doi: 10.3390/cancers11101461 (PMC6826833; doi:10.3390/cancers11101461)
Supplement: Supplementary file 1 [file cancers-11-01461-s001.zip › cancers-565117-supplementary-final.pdf]

Supplementary Materials: Arl13b Regulates Breast Cancer Cell Migration and Invasion by Controlling Integrin-Mediated Signaling

En Cristina Casalou, Alexandra Faustino, Fernanda Silva, Inês C. Ferreira, Daniela Vaqueirinho, Andreia Ferreira, Pedro Castanheira, Teresa Barona, José S. Ramalho, Jacinta Serpa, Ana Félix and Duarte C. Barral

Methods

Trypan Blue Exclusion Test

Control (shRNA Empty and shRNA Mission) and shRNA Arl13b (E4 and E6) MDA-MB-231 cells (4.5–5 × 10^4) were plated in 24-well plates in DMEM complete medium. After 24 and 48 hours, cell number was determined using a hemocytometer. To control for the effect of mitomycin C on cell proliferation, 8.5 × 10^4 MDA-MB-231 cells silenced for Arl13b (shRNA E4 and E6) and control cells (shRNA Empty and shRNA Mission) were serum-starved, treated with 2.5 μg/mL of mitomycin C for 2 hours and then grown in complete medium. After 21–24 hours, cell number was determined as before. In both cases, trypan blue staining was used to exclude dead cells.

Immunohistochemistry

Normal breast tissues from mammoplasty and invasive ductal breast tumor paraffin-embedded samples were obtained from the tumor bank of the Pathology Department of Hospital Beatriz Ângelo (HBA). The study was approved by the ethics committee of HBA (Ref. #0174). Paraffin blocks were deparaffinized, endogenous peroxidase was blocked with 3% H_2O_2 and antigen retrieval was performed using a pressure cooker in citric acid (pH = 6.0) with 0.05% Tween-20. Unspecific binding was blocked with 0.1% BSA for 1 hour and the slides were washed in PBS with 0.05% Tween-20 and incubated with anti-Arl13b antibody (Proteintech) in Dako REAL antibody diluent (Dako) overnight at 4 °C in a humidified chamber. After washing in PBS/0.05% Tween 20, slides were incubated with anti-rabbit-HRP secondary antibody (Jackson ImmunoResearch) in Dako REAL antibody diluent for 1 hour at RT.

Time-lapse fluorescence microscopy

MDA-MB-231 cells grown on 8-well lab-Tek chambered #1.0 borosilicate coverglass system (Thermo Scientific Nunc) coated with 10 μg/mL fibronectin in DMEM 10% FBS were transfected with Arl13b-EGFP- and paxillin-DsRed-encoding vectors using Lipofectamine 2000 (Invitrogen) according to the manufacturer's instructions. Paxillin-DsRed construct was a kind gift of J. Casanova (University of Virginia School of Medicine, Virginia, USA) and Arl13b-EGFP of K. Kontani (University of Tokyo, Tokyo, Japan) [1]. Twenty-four hours after transfection, the medium was changed to DMEM without phenol red, supplemented with 10% FBS, 100 U/mL penicillin, 100 μg/mL streptomycin, 2 mM L-glutamine, 1 mM sodium pyruvate and 30 mM HEPES and co-transfected cells were imaged for 3–10 minutes at 5–10 second intervals using a Zeiss LSM 710 confocal microscope equipped with a Plan-Apochromat 63/1.40 Oil Ph3 lens and Zen Blue 2010b SP1software.

Supplementary Figure and Tables

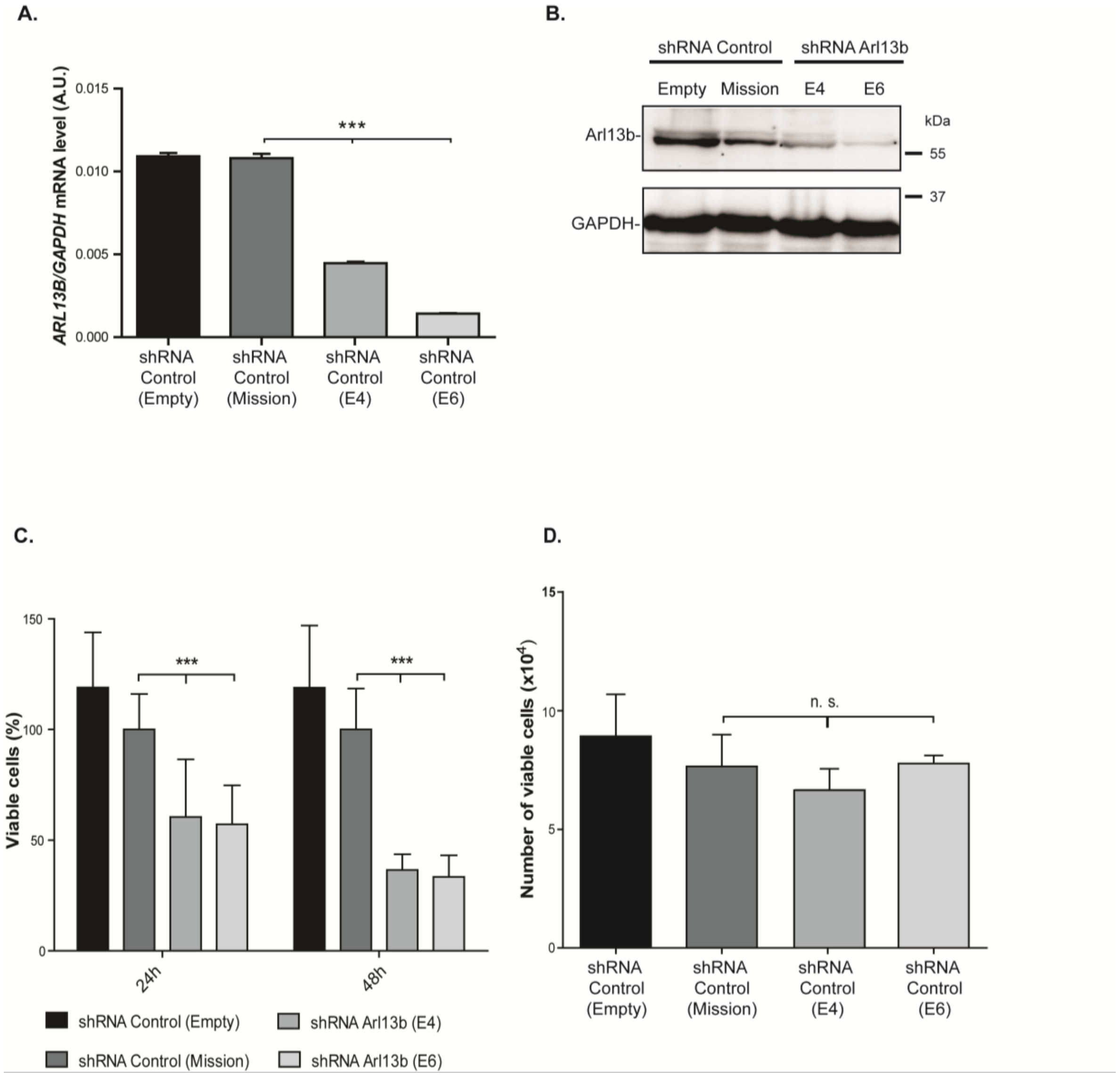

Figure S1. Arl13b silencing efficiency and effect on cell growth. Arl13b silencing using two distinct *ARL13B*-targeting shRNAs (E4 or E6) was determined by RT-qPCR (**A**) and by immunoblotting (**B**). (**A**) Representative plot of *ARL13B* mRNA levels in Arl13b-silenced (shRNA E4 and E6) and control (shRNA Empty and shRNA Mission) cells, measured relative to the housekeeping gene *GAPDH*. *** $p < 0.001$ (unpaired two-tailed Student's *t*-test). A.U., arbitrary units (**B**) Representative immunoblot analysis of Arl13b protein levels in Arl13b-silenced and control cells. (**C**) Cell growth of Arl13b-silenced and control cells at 24 and 48 hours after plating, determined by trypan blue exclusion. For each condition, the number of viable cells was normalized to shRNA control (Mission), in which viability was considered 100%. *** $p < 0.001$ (two-way ANOVA). (**D**) To block cell proliferation during migration and invasion assays, serum-starved Arl13b-silenced and control cells were pre-treated with 2.5 μg/mL mitomycin C for 2 hours. The effect of mitomycin C was evaluated by counting the number of cells at 21-24 hours after plating. Dead cells were excluded by trypan blue staining. Error bars represent mean ± SD ($n = 3$). n.s., non-significant.

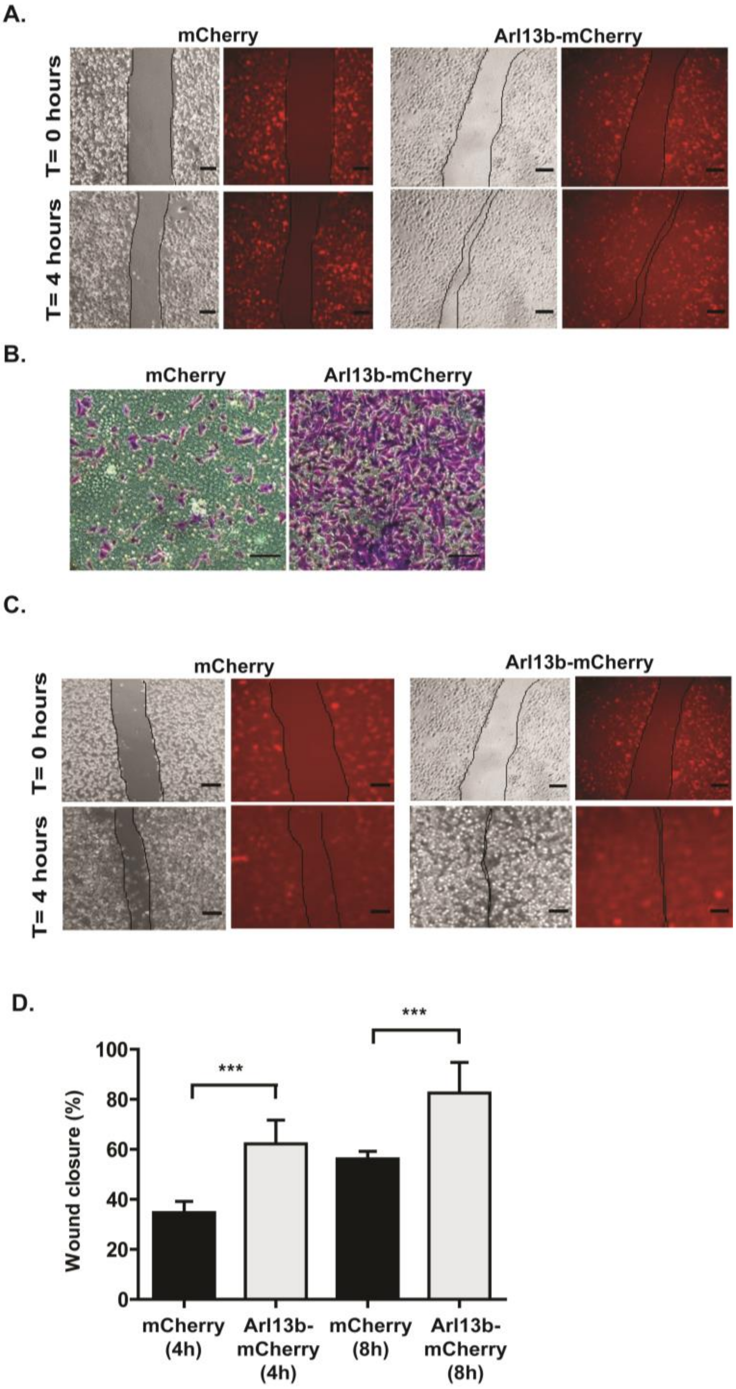

Figure S2. Arl13b overexpression enhances migration and invasion of breast cancer cells. (**A**) MDA-MB-231 cells transduced with lentiviruses encoding Arl13b-mCherry or mCherry (control) were grown to confluency on a monolayer, scratched with a pipette tip and gap ("wound") closure monitored for 21 hours in medium with 10% FBS. Representative images taken at 0 and 4 hours are shown. Scale bars, 20 µm. (**B**) MDA-MB-231 cells overexpressing Arl13b-mCherry or mCherry were placed in serum-free medium into the upper chamber of a transwell coated with matrigel and induced to invade and migrate towards 10% FBS medium, added to the lower chamber. Cells that invaded were fixed and stained with crystal violet. Representative images are shown. Scale bars, 50 µm. (**C**) MCF-7 cells transduced as in (**A**), grown to confluency were stimulated to migrate after a scratch with a pipette tip. Representative images taken at 0 and 4 hours are shown. Scale bars, 20 µm. (**D**) The area between the gap edges was measured at 0, 4 and 8 hours to determine the percentage of gap ("wound") closure. Error bars represent the mean ± SD (n = 3). *** $p < 0.001$ (unpaired two-tailed Student's t-test, Mann-Whitney).

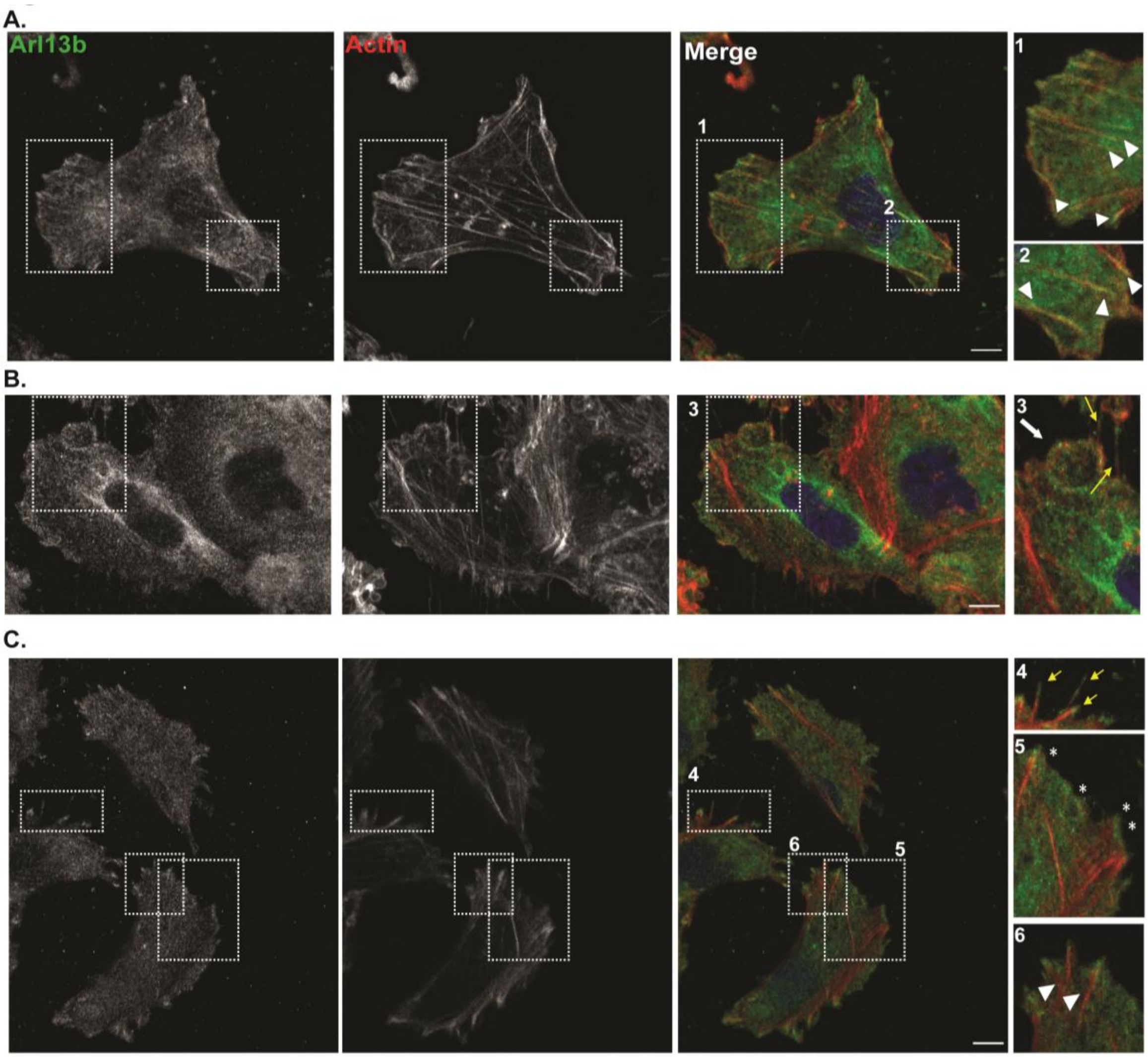

Figure S3. Arl13b localizes to actin-rich structures in breast cancer cells. MDA-MB-231 cells were immunostained with anti-Arl13b antibody (green) and co-stained with phalloidin to visualize F-actin (red) and DAPI (blue) to visualize nuclei. Cells were analyzed by confocal microscopy. Zoom-ins of the indicated sections are shown in the insets. (**A**,**C**) Arrowheads point to Arl13b localized to stress fibers (zoom-ins 1, 2 and 6). (**B**) White arrow indicates Arl13b localized to a circular dorsal ruffle (zoom-in 3). (B, C) Yellow arrows indicate Arl13b localized to filopodia (zoom-ins 3 and 4). (C) White stars indicate Arl13b localization to structures consistent with lamellipodia (zoom-in 5). Scale bars, 10 µm.

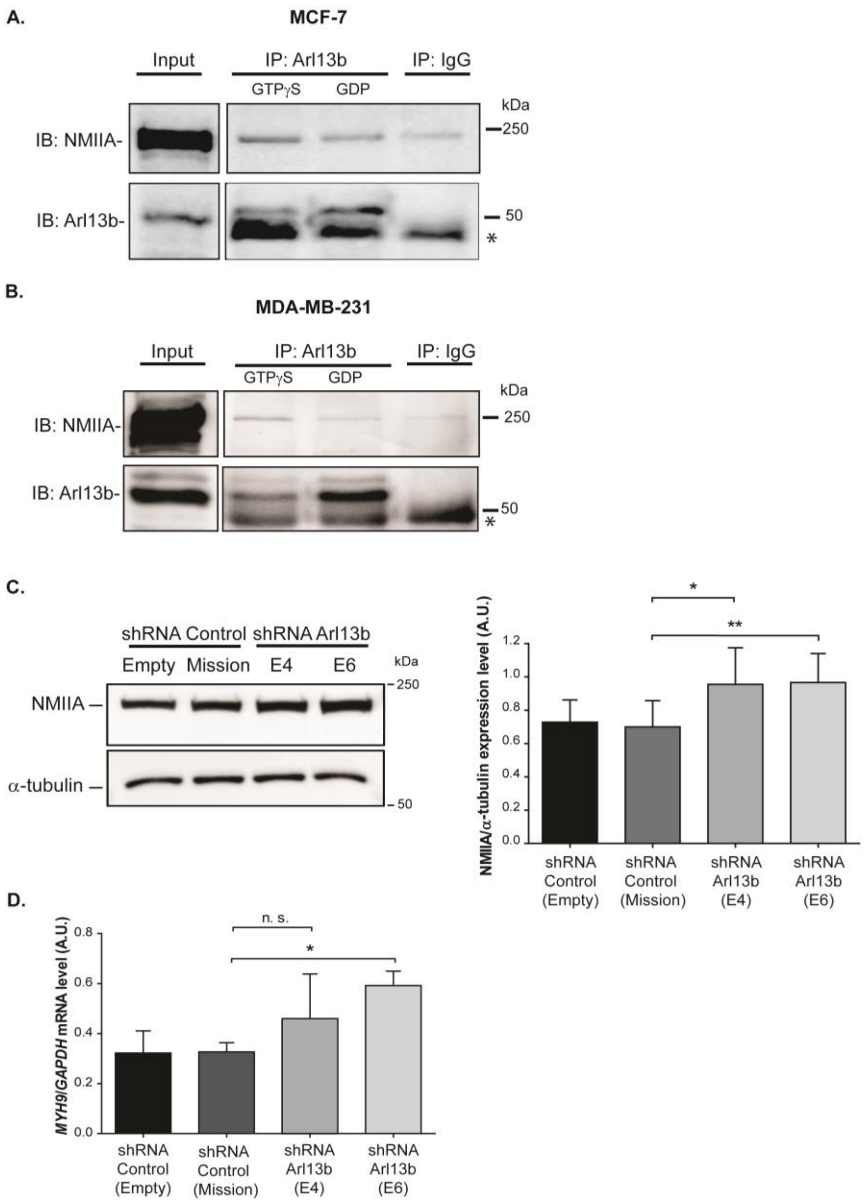

Figure 4. Arl13b regulates the expression of Myosin IIA and interacts with it in breast cancer cells, preferentially in the active state. Lysates from MCF-7 (**A**) and MDA-MB-231 (**B**) cells were immunoprecipitated (IP) with anti-Arl13b antibody in the presence of GTPγS or excess GDP. As a negative control, rabbit IgG in the presence of GTPγS was used. Immunoprecipitated products were separated by SDS-PAGE and immunoblotted (IB) with antibodies against the indicated proteins. Experiments were independently performed twice. *, heavy chain of the antibody used for the immunoprecipitation. (**C**) NMIIA protein levels were determined by immunoblotting in total cell extracts of Arl13b-silenced (shRNA E4 and E6) or control (shRNA Mission and Empty) MDA-MB-231 cells grown on wells coated with 10 μg/mL fibronectin in DMEM with 10% FBS. NMIIA expression levels were normalized to α-tubulin levels, used as loading control. Error bars represent mean ± SD ($n \geq 5$). * $p < 0.05$, ** $p < 0.01$ (unpaired two-tailed Student's *t*-test, Mann-Whitney). (**D**) *MYH9* (NMIIA) mRNA levels were determined in Arl13b-silenced (shRNA E4 and E6) and control (shRNA Empty and shRNA Mission) MDA-MB-231 cells. *GAPDH* was used as housekeeping gene. * $p < 0.05$; n.s., non-significant (unpaired two-tailed Student's *t*-test, Mann-Whitney). A.U., arbitrary units.

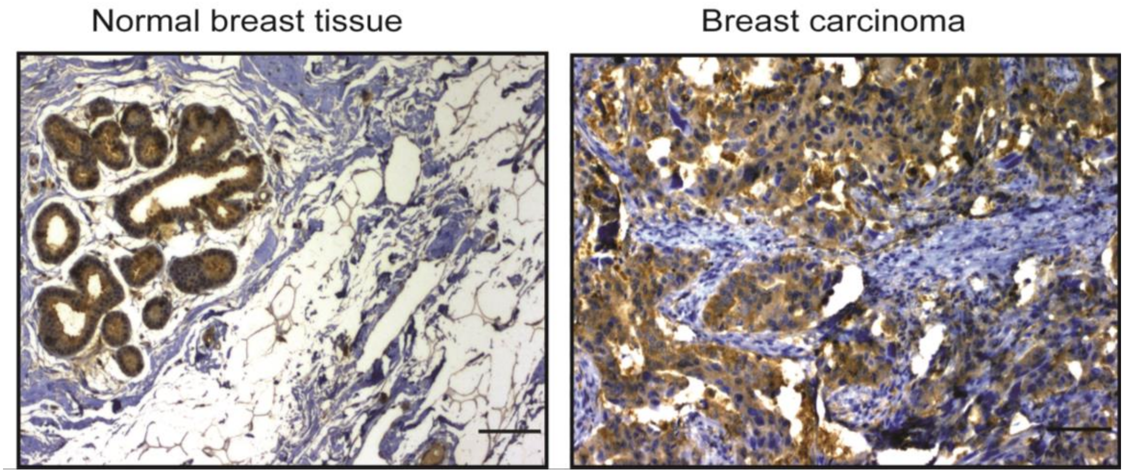

Figure S5. Arl13b expression in normal breast tissue and in breast carcinoma. Arl13b expression, as determined by immunohistochemistry, in an invasive breast carcinoma (62 years old patient, stage III, 15 mm, T1c, N0, G3) and normal breast tissue derived from mammary surgical reduction. Positive staining is observed in normal ductal epithelial cells and tumor cells. Scale bars, 100 μm.

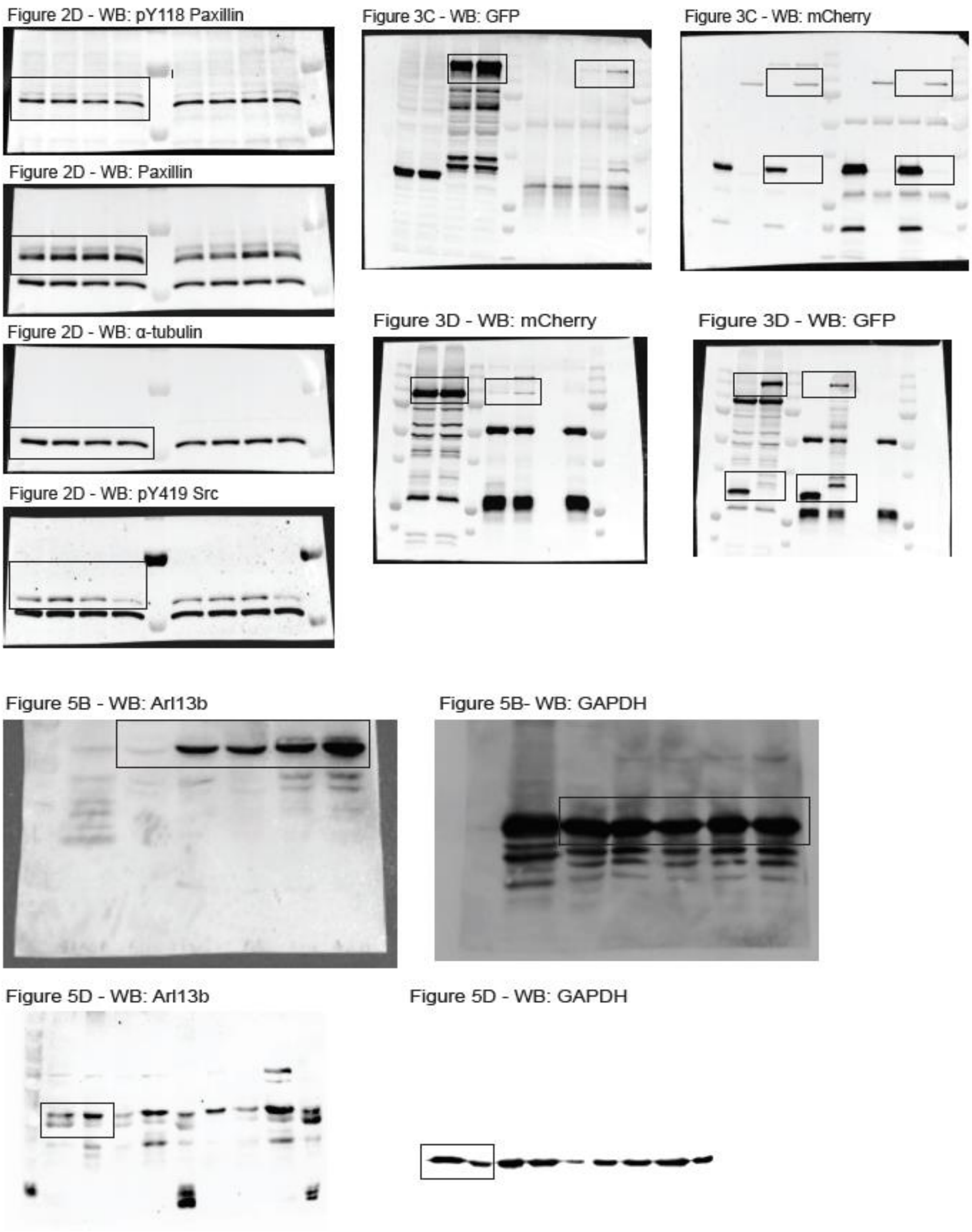

Figure S6. Uncropped immunoblots of main text Figures.

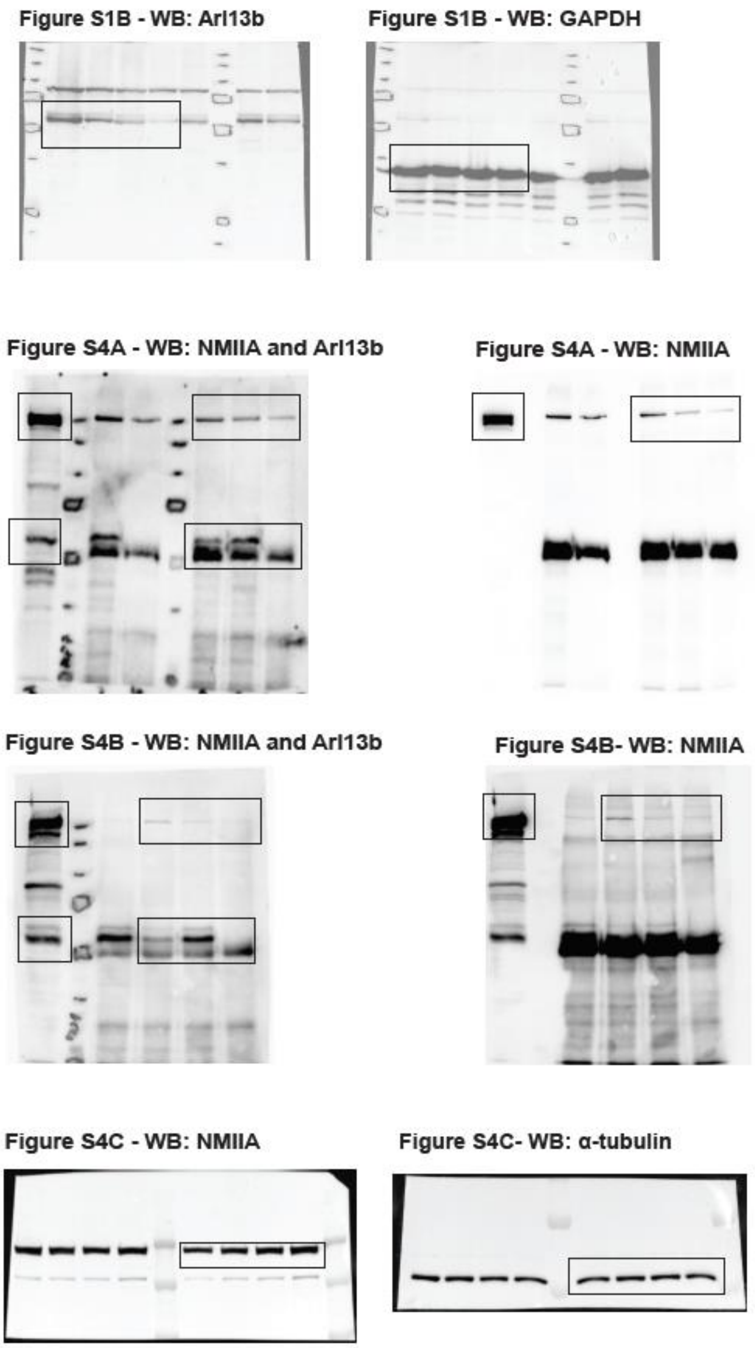

Figure S7. Uncropped immunoblots of supplementary Figures.

Table S1. Primer sequences used for RT-qPCR.

Gene	Primer sequence (5′-3′)
ARL13B	GAATCCAAGGAGAATACCCTG
	CCAACACCAATATAGGCTTTCC
MYH9	AGAAGGTGAAGGTGAACAAGG
	GGTGTAGATGAGCCCTGAG
GAPDH	CATTTCCTGGTATGACAACGA
	GTCTACATGGCAACTGTGAG

Table S2. Concentrations and dilutions of antibodies and dyes.

Antigen (Clone)	Brand and Catalog #	IB (µg/mL)	FC (µg/mL)	IF (µg/mL)	IP (µg)	IHC (µg/mL)
**Arl13b**	Proteintech # 17711	0.14–0.27		2.73		5.46
**Arl13b**	Abcam # ab136648	5				
**Arl13b**	[2]	4				
**Arl13b**	Sigma-Aldrich # HPA048926					0.2
**GFP**	SicGen # AB0020	2			3	
**GFP**	[3]	[1:2 500]				
**mCherry**	SicGen # AB0040	3			3	
**β3-integrin**	Santa Cruz # sc-20058		4			
**GAPDH**	SicGen # AB0049	0.4				
**α-tubulin**	Sigma-Aldrich # T6199	0.1				
**Paxillin (5H11)**	Invitrogen # MA5-13356	0.5				
**Phospho-Paxillin**	Invitrogen # 44-722G	[1:500]				
**Myosin IIA**	Sigma-Aldrich #M8064	0.1–0.7				
**Vinculin**	Sigma-Aldrich #V9264			5		
**Phospho-Src**	Santa Cruz # sc-101802	0.67				
**HRP-conjugated anti-rabbit**	GE Healthcare #NA934	[1:5 000]				
**HRP-conjugated anti-mouse**	GE Healthcare # NA931	[1:5 000]				
**HRP-conjugated anti-goat**	Jackson Immuno-Research #705-035-147	0.1				
**HRP-conjugated IgG fraction anti-rabbit**	Jackson Immuno-Research #211-032-171	0.4				1.6
**Alexa Fluor 488-anti-mouse**	Invitrogen #A-11001		10	5		
**Alexa Fluor 488-anti-rabbit**	Invitrogen #A-21206			2		
**Alexa Fluor 568-Phalloidin**	Invitrogen # A12380			0.03		

IB—Immunoblotting; FC—Flow cytometry; IF—Immunofluorescence; IP—Immunoprecipitation; IHC—Immunohistochemistry; HRP—Horseradish peroxidase.

Table S3. Clinicopathological characteristics of invasive ductal breast carcinomas obtained from the tumor bank of the pathology department of Hospital Beatriz Ângelo (HBA).

Sample Number	Age at Diagnosis (years)	Stage (AJCC 2017)	Tumor Size (mm)	Tumor	Node	Histological Grade
1	70	IA	15	T1c	N0	G1
2	51	IIA	30	T2	N1a	G1
3	57	IB	35	T2	N0	G1/2
4	77	IA	4	T1a	N0	G1
5	70	IB	25	T2	N0	G2
6	57	IA	20	T1c	N0	G2
7	65	IA	20	T1(m)	N0	G2
8	46	IB	20	T1c	N1a	G2
9	72	IB	25	T2	N0	G2
10	68	IA	16	T1c	N0	G2
11	64	IA	12	T1b	N0	G2
12	74	IIA	20	T2	N1	G2
13	64	IIA	28	T2	N1a(sn)	G2
14	62	IA	8	T1b	N0	G2

T—Tumor; N—Node; (m) —Multifocal tumors; (sn) —Sentinel node

Table S4. Clinicopathological characteristics of breast carcinomas obtained from the tumor bank of the pathology department of Instituto Português de Oncologia de Lisboa Francisco Gentil (IPOLFG).

Sample Number	Age at Diagnosis (years)	Stage (AJCC 2017)	Tumor Size (mm)	Tumor	Node	Histological Grade
1	49	IIA	35	T2	N0	G2
2	47	IIB	30	T2	N1	G2
3	47	IIA	22	T2	N0	G1
4	64	IA	16	T1c	N0	G3
5	56	IIA	28	T2	N1a	G3
6	64	IB	20	T1c	N0	G2
7	56	IIA	26	T2a	N0	G3
8	74	IIA	35	T2	N0	G3
9	46	IA	23	T1c	N0	G3
10	42	IIIA	30	T2 (m)	N2a	G3
11	45	IA	16	T1c	N0	G2
12	72	IIA	17	T1c	N1a	G3
13	69	IA	13	T1c	N0	G3
14	32	IA	13	T1	N0(sn)	G3
15	53	IA	20	T1c (m)	N0	G2
16	85	IIB	35	T2	N1a	G3
17	40	IIA	22	T2	N0(sn)	G2
18	43	IB	17	T1c	N1a	G2
19	49	IIB	25	T2	N1a	G3
20	68	IIA	18	T1c	N1a	G2
21	78	IA	13	T1c	N0	G2
22	47	IA	20	T1c	N0	G2
23	54	IIIA	50	T3	N1a	G2
24	49	IIA	30	T2	N0	G2
25	81	IIIC	26	T2	N3a	G3
26	38	IIB	45	T2	N1a	G3
27	66	IIB	32	T2(m)	N1a	G3
28	62	IA	13	T1c	N0	G2
29	66	IIB	35	T2	N1a	G3
30	63	IIA	15	T1c	N1(mi)	G3
31	69	IA	18	T1c	N0	G2
32	53	IIB	22	T2	N1	G2

T—Tumor; N—Node; (m) —Multifocal tumors; (sn)—Sentinel node; (mi)—Micrometastasis (<0.2 mm)

Video S1. Arl13b localizes to focal adhesions. MDA-MB-231 cells co-transfected with Arl13b-EGFP and Paxilin-DsRed were analyzed for 3 minutes at 5 second intervals. Scale bar, 10 µm.

References

1. Hori, Y.; Kobayashi, T.; Kikko, Y.; Kontani, K.; Katada, T. Domain Architecture of the Atypical Arf-Family GTPase Arl13b Involved in Cilia Formation. *Biochem. Biophys. Res. Commun.* **2008**, *373* (1), 119–124. https://doi.org/10.1016/j.bbrc.2008.06.001.
2. Barral, D. C.; Garg, S.; Casalou, C.; Watts, G. F. M.; Sandoval, J. L.; Ramalho, J. S.; Hsu, V. W.; Brenner, M. B. Arl13b Regulates Endocytic Recycling Traffic. *Proc. Natl. Acad. Sci. U. S. A.* **2012**, *109* (52), 21354–21359. https://doi.org/10.1073/pnas.1218272110.
3. D'Angelo, R.; Aresta, S.; Blangy, A.; Del Maestro, L.; Louvard, D.; Arpin, M. Interaction of Ezrin with the Novel Guanine Nucleotide Exchange Factor PLEKHG6 Promotes RhoG-Dependent Apical Cytoskeleton Rearrangements in Epithelial Cells. *Mol. Biol. Cell* **2007**, *18* (12), 4780–4793. https://doi.org/10.1091/mbc.e06-12-1144.

© 2019 by the authors. Licensee MDPI, Basel, Switzerland. This article is an open access article distributed under the terms and conditions of the Creative Commons Attribution (CC BY) license (http://creativecommons.org/licenses/by/4.0/).
